# Special Issue: Innovative Pasta with High Nutritional and Health Potential

**DOI:** 10.3390/foods11162448

**Published:** 2022-08-14

**Authors:** Laura Gazza, Francesca Nocente

**Affiliations:** CREA-Research Center for Engineering and Agro-Food Processing, Via Manziana 30, 00189 Rome, Italy

This editorial summarizes some of the key challenges in the production of novel pasta formulations in order to obtain high nutritional and healthy products. In the last 20 years, the awareness of the effect of nutrition on human health increased the interest of consumers in food with enhanced nutritional characteristics. Consequently, the agro-food sector was prompted towards the research and development of functional foods presenting healthy features that go beyond their nutritional value. This new trend also involved the pasta sector to look for innovative pasta formulations by replacement or enrichment of durum wheat semolina with functional ingredients from plant [1,2,3] or animal origin [2,4,5], or by the use of alternative raw materials such as gluten-free cereals [4,6,7], minor cereals [3,8,9], pseudocereals and legumes [2]. Indeed, pasta, due to its versatility, low cost, nutritional value and pleasant taste, is largely consumed worldwide, thus representing a suitable carrier to deliver functional molecules exerting human-health-beneficial effects. The use of functional ingredients or alternative raw materials often change the cooking quality and sensory attributes of pasta, resulting in increased cooking loss, decreased firmness, color changes and pasta flavor. Consequently, the strategy of pasta innovation, along with the identification of functional ingredients, required suitable technological process to obtain novel pasta products, able to retain their beneficial properties, keeping, meanwhile, high cooking and sensorial quality to be attractive to consumers [3,6,10,11]. Dried pasta is the symbol of Italian food, and it is certainly no coincidence that 7 out of the 11 articles published in this Special Issue, come from Italian research groups [2,3,6,7,8,10,11]. Pasta functionalization has been mainly reached by fortification with dietary fiber [3,6], antioxidants [4,5], proteins [2], vitamins and minerals [1], which are present in a limited amount in durum wheat semolina. The consumption of high-fiber pasta contributes to reducing blood pressure, cholesterol levels, risk of colon cancer and coronary heart disease and improving the feeling of satiety. Moreover, the enrichment with dietary fiber also reduces the glycemic index of pasta, a health aspect often considered in this Special Issue [6,7,8,10,11]. Generally, the presence of dietary fiber results in detrimental effects on the cooking and sensory qualities of pasta, although the source of fiber used and the level of enrichment, as well as the technological process applied, largely influence the overall quality of unconventional pasta products. The technological procedures useful to overcome the negative effect of wheat bran enrichment include micronization [3,6], air classification, debranning, fermentation [10] and enzymatic hydrolysis. Besides the presence of dietary fiber, the importance of wholegrain consumption is also related to the presence of health-promoting antioxidant compounds, mostly present in the outer kernel layers. Polyphenols are the most abundant plant antioxidants and the long-term consumption of foods rich in polyphenols is considered healthier by reducing the risk of some types of cancer, diabetes and neurodegenerative and cardiovascular diseases. Fruits and vegetables are particularly rich in polyphenols as well as vitamins, minerals and fiber. Hence, they have been used as ingredients mainly to enhance the antioxidant activity of pasta by enrichment with flour from dried fruit and vegetables [1,2,3], with pureed or with antioxidant compounds extracted from them. Recently, pasta functionalization has also been realized by the use of novel ingredients such as edible insects, fish [4,5], seaweed [2] and meat as a source of proteins, fiber, vitamins, minerals and beneficial fats. For example, the partial replacement of semolina with cricket flour increase proteins, fiber, unsaturated fatty acids, minerals and antioxidant activity, while it decreases carbohydrate content of enriched pasta. Moreover, the growing demand for gluten-free products for people affected by celiac and non-celiac gluten sensitivity and for consumers who prefer a gluten-free diet has been satisfied by the presence on the market of a broad range of high-quality gluten-free pasta. Although the lack of gluten makes the processability of dough into pasta very difficult, the replacement of semolina with gluten-free cereals (rice, corn, sorghum), pseudocereals (quinoa, chia, buckwheat), and legumes (beans, fava beans, peas, chickpeas, lentils, lupins) and the use of additives (gums, emulsifiers or hydrocolloids) and appropriate technologies has allowed industries to produce gluten-free pasta with acceptable sensorial characteristics [4,6,7].

Finally, the pasta sector does not overlook the transition towards a circular economy, one of the European Green Deal pillars, essential for the sustainability of food systems in terms of reducing both resource consumption and waste into the environment. These issues can be addressed by the re-use of food by-products and food wastes to increase the nutritional value of pasta, being often a good source of proteins, minerals, fatty acids, fiber and bioactive compounds. Four articles of this Special Issue reported the results of the up-cycling of fruit and vegetable by-products [1,3] or fish processing [4,5] by the production of dry pasta, with an increased potential nutritional value, as an example of “circular” innovation in the pasta food chain.
